# Walking, orthoses and physical effort in a Swedish population with arthrogryposis

**DOI:** 10.1007/s11832-014-0597-9

**Published:** 2014-06-08

**Authors:** Marie Eriksson, Li Villard, Åsa Bartonek

**Affiliations:** 1 Department of Women’s and Children’s Health, Karolinska Institutet, Stockholm, Sweden; 2 Department of Physiotherapy, Astrid Lindgren Children’s Hospital, Stockholm, Sweden; 3 Q2:07 MotorikLab, Astrid Lindgren Children’s Hospital, 171 76 Stockholm, Sweden

**Keywords:** Ambulation, Amyoplasia, Oxygen consumption, Oxygen cost, Physical capacity

## Abstract

**Purpose:**

Excessive movements during walking have been observed by gait analysis in children with arthrogryposis (AMC) using orthoses compared to children using only shoes. The aim of this study was to evaluate energy expenditure and functional exercise capacity in children with AMC.

**Methods:**

Twenty-four children with AMC and 25 typically developing (TD) children underwent oxygen measurement and the 6-minute walk test (6MWT). Children were divided into AMC1 using knee–ankle–foot orthoses with locked knee joints (KAFO-LK); AMC2 KAFOs with open knee joints (KAFO-O) or ankle–foot orthoses (AFO); and AMC3 using shoes.

**Results:**

The net non-dimensional oxygen cost (NNcost) was lower in TD (0.308) than in AMC2 (0.455, *n* = 10) (*p* = 0.002). There were no differences in the net non-dimensional consumption (NNconsumption) or normalised walking velocity. The lowest NNconsumption (0.082), NNcost (0.385) and normalised walking velocity (0.214) were found in AMC1 (*n* = 3), but no statistical calculation was performed. In the 6MWT, both AMC2 (402.7, *n* = 11) and AMC3 (476.8, *n* = 10) walked shorter distances (m) than TD (565.1) (*p* < 0.001 and *p* = 0.043, respectively). AMC2 (0.435) had lower normalised walking velocity than TD (0.564) (*p* < 0.001).

**Conclusions:**

Children with AMC using open KAFOs or AFOs (AMC2) had higher energy effort represented by significantly higher NNcost than TD, whereas AMC children requiring only shoes (AMC3) did not differ significantly from TD. To maintain the NNconsumption at an acceptable level, children using locked KAFOs (AMC1) slowed down their walking velocity. Compared to TD, the exercise capacity was lower in children with AMC using open KAFOs or AFOs and shoes, represented by lower walking velocity and shorter distance walked during the 6MWT.

## Introduction

Arthrogryposis (AMC) is characterised by the presence of multiple joint contractures in multiple body areas that are present at birth [1], with a reported incidence from 1 per 3,000 to 1 per 5,100 live births [1, 2]. It has been defined as a rare but heterogeneous disorder [3] with different subtypes, of which amyoplasia is the most common [1]. The ability of functional ambulation depends on factors such as the severity of lower limb deformities and muscle weakness in the lower limbs, primarily in the hip and knee extensor muscles [4, 5]. Independent walking before the age of 2.5 years has been reported [5], and 85 % of children with amyoplasia were found to be ambulators by the age of 5 years [6]. The presence of muscle weakness in the lower extremities was assumed to have more influence on walking ability than the severity of contractures [7]. For efficient mobility, the use of a wheelchair may be required [3, 6, 8].

Orthopaedic surgery with multiple procedures is often necessary to achieve functional ambulation [3]. Orthoses are also often used to enhance or facilitate walking and to compensate for muscle weakness and lower extremity deformities, as well as to maintain the lower extremities in an aligned position [4, 9]. The most commonly used orthoses types in children with AMC are ankle–foot orthoses (AFOs) or knee–ankle–foot orthoses (KAFOs) with a knee locking mechanism [4, 9]. In children with plantarflexor weakness, orthoses with a carbon fibre spring ankle joint were considered to improve gait, as measured by 3-D gait analysis [10]. Variations in gait pattern with respect to orthosis use have been observed; in children using KAFOs with locked knee joints, more extensive trunk and pelvic movements were observed compared to children using AFOs and shoes [11]. It may, thus, be assumed that physical effort during walking is high in children with AMC.

Various methods have been proposed to assess physical effort during walking. A functional exercise capacity test has been developed for use during a 6-min walking period that reflects the functional level for daily physical activities [12]. The physiological cost index (PCI) offers a value of the heart rate per metre walked [13]. Although assuming a linear relation between heart rate and oxygen consumption [14], the PCI method has been criticised for use in children with disabilities, due to the children’s difficulties in achieving a state of steady heart rate during walking [15, 16]. Instead, the measurement of oxygen uptake has been recommended, allowing calculation of the oxygen consumption and cost, referred to as physical effort and indicator of the efficiency of walking, respectively [17]. To reduce the variability between participants of different ages, a non-dimensional normalisation scheme for oxygen data has been proposed [18]. The scheme uses the net oxygen utilisation instead of gross oxygen utilisation, the former evaluating solely the amount of energy used to ambulate except resting time [18].

In youths with AMC, the ambulatory and physical activity has been studied using a step activity monitor and was found to be lower compared to that of typically developing (TD) youths [19]. To our knowledge, there is no report on energy effort; thus, the aim of this study was to evaluate energy expenditure and exercise capacity during walking in children with AMC.

## Methods

### Participants

Children with AMC born between the years 1993 and 2007, who were treated at the children’s orthopaedic department of Karolinska University Hospital, Stockholm and at the Uppsala University Children’s Hospital, were invited to participate in the study. Thirty-one children fulfilled the inclusion criteria of lower limb deformities or contractures, independent ambulation with or without orthoses, and age between 5 and 18 years. None of the children had undergone orthopaedic surgery in the past 12 months. Of the 31 children eligible for inclusion, seven families declined participation, thus, 24 children with a mean age of 11.1 (4.3) years took part in the study between April 2011 and March 2012. Twenty-five TD children with a mean age of 11.4 (4.1) years constituted the control group. The study was approved by the Regional Ethical Review Board in Stockholm, Sweden. Written informed consent was obtained from the participants and their parents.

**Fig. 1:**
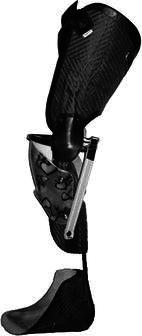
Knee–ankle–foot orthosis with locked knee joint and carbon fibre spring ankle joint (KAFO-LK-C)

**Fig. 2:**
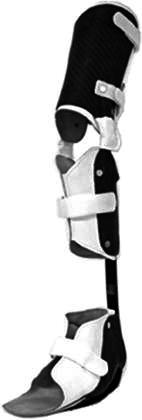
Knee–ankle–foot orthosis with open knee joint and extension stop, and carbon fibre spring ankle joint (KAFO-O-C)

**Fig. 3:**
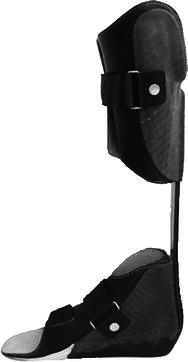
Ankle–foot orthosis with carbon fibre spring ankle joint (AFO-C)

The children were divided into three groups based on orthosis use, as has been described in a previous study [11]: AMC1 (*n* = 3) wore KAFOs with locked knee joints (KAFO-LK); in AMC2 (*n* = 11), 3/11 children wore KAFOs with open knee joints (with an extension stop) (KAFO-O), 7/11 wore AFOs of different types and 1/11 wore KAFO-LK with compensation for limb length discrepancy and a foot orthosis (FO); and AMC3 (*n* = 10) used shoes. The distributions of gender, age, height and weight are shown in Table 1. Prescriptions of orthoses were based on the presence of muscle weakness, joint contractures and deformities according to the orthotic programme of Karolinska University Hospital. In AMC1, the locked knee joints were prescribed due to knee extensor weakness. In this group, two children used carbon fibre springs (Fig. 1). In AMC2, KAFO-Os with free flexion were prescribed in one child with hyperextension and in two children to control foot and thigh alignment, all of them with carbon fibre springs (Fig. 2). AFOs with carbon fibre springs (Fig. 3) were prescribed in two children with plantarflexor weakness. Five children had various AFO types (two solid, two hinged and one flexible carbon fibre) to stabilise the foot and ankle joint based on material criteria due to weight and acceptance of orthoses (Table 2). All children used their orthoses for 8 h or more daily.

**Table 1:** Distributions of gender, age, height and weight mean (SD) in the groups

	AMC1	AMC2	AMC3	TD
Gender				
Male	3	9	4	17
Female	–	2	6	8
Age (years)	16.8 (0.2)	10.0 (4.1)	10.7 (4.1)	11.4 (4.1)
Height (cm)	169.0 (3.5)	132.6 (22.8)	135.8 (25.2)	148.8 (24.1)
Weight (kg)	70.7 (27.0)	29.9 (12.0)	35.3 (14.9)	43.6 (18.0)

*AMC* arthrogryposis multiplex congenita, *AMC1* used knee–ankle–foot orthoses with locked knee joints, *AMC2* used knee–ankle–foot orthoses with open knee joints or ankle–foot orthoses, *AMC3* used shoes, *TD* typically developing children

**Table 2:** Orthoses types, group, functional ambulation, muscle strength, joint contractures and previous orthopaedic surgery in all participants with AMC

Subject	Orthoses	Group	Functional ambulation	Muscle strength (grade 0–5)					Joint contractures (º)			Orthopaedic surgery procedure	
				Hip		Knee	Ankle		Hip	Knee	Plantar	Bony	Soft tissue
				Ext L/R	Abd L/R	Ext L/R	Dors L/R	Plant L/R	Flexion L/R	Flexion L/R	Flexion L/R		
1	KAFO-LK-C	AMC1	HA	4/4	4/4	3/3	0/0	0/0	–	20/+15	40/50	Hip bi, ankle bi	–
2	KAFO-LK	AMC1	HA	4/4	4/4	3/3	0/0	0/0	–	–/10	20/20	Knee bi, ankle bi	–
3	KAFO-LK-C	AMC1	HA	4/4	4/4	3/3	1/1	1/1	–	30/30	25/25	Knee bi, ankle bi	–
4	KAFO-O-C	AMC2	HA	4/4	4/4	4/3	0/0	0/1	–	10/–	–/10	Knee uni, ankle bi	Knee uni
5	AFO-H	AMC2	CA	4/4	4/4	4/4	3/4	4/4	–	+15/–	–/5	–	Ankle bi
6	AFO-S	AMC2	HA	4/4	4/4	4/4	3/3	1/1	–	25/20	–	Ankle bi	–
7	KAFO-O-C	AMC2	CA	4/4	4/4	4/4	0/0	0/0	–	10/10	15/5	Hip uni, ankle bi	–
8	AFO-H	AMC2	CA	4/4	4/4	4/4	4/4	2/2	–	+20/+20	–	Ankle bi	–
9	AFO-C^a^	AMC2	CA	5/4	5/4	5/4	4/4	4/3	–	–/10	–/10	–	Knee uni, ankle uni
10	KAFO-O-C	AMC2	CA	5/5	5/5	5/5	4/4	4/4	–	+20/+20	10/–	Ankle bi	–
11	AFO-S^a^	AMC2	CA	4/4	4/4	5/5	5/3	5/3	–	–	–	Spine fusion	Ankle uni
12	AFO-C	AMC2	CA	4/4	4/4	5/5	4/4	3/3	–	–	–	Ankle bi	–
13	FO/KAFO-LK^b^	AMC2	CA	5/4	5/4	5/0	5/0	5/0	–/15	–/75	10/50	Knee uni	–
14	AFO-FC	AMC2	CA	4/4	4/4	4/4	2/2	2/2	–	–	10/–	Ankle bi	–
15		AMC3	CA	4/4	4/4	4/4	4/4	4/4	10/–	–	–	Ankle bi	–
16	Insoles	AMC3	CA	4/4	4/4	4/4	4/4	4/4	20/20	–	15/–	–	–
17		AMC3	CA	4/4	4/4	4/4	4/3	4/4	–	10/–	–	–	Knee bi, ankle uni
18		AMC3	CA	4/4	5/5	5/5	4/4	4/4	10/10	–/+20	–	Ankle bi	–
19		AMC3	CA	5/5	5/5	5/5	3/3	4/4	–	–	–	–	–
20		AMC3	CA	4/4	4/4	4/4	4/4	4/4	–	+20/+15	–	Hip bi, ankle uni	–
21	Insoles	AMC3	CA	4/4	4/4	4/4	3/4	4/4	–	–	–	Hip bi	–
22		AMC3	CA	5/5	5/5	4/4	4/4	4/4	–	–	–	Knee bi	Ankle uni
23		AMC3	CA	5/5	5/5	4/4	4/4	4/4	–/10	–	–	Spine fusion	–
24		AMC3	CA	5/5	5/5	4/4	4/4	5/5	–/10	–	5/–	Hip bi	–

*KAFO* knee–ankle–foot orthosis, *LK* locked knee joint, *C* carbon fibre ankle joint, *O* open knee joint with extension stop, *AFO* ankle–foot orthosis, *H* hinged, *S* solid, *FO* foot orthosis, *FC* flexible carbon fibre, *AMC* arthrogryposis multiplex congenita, *HA* household ambulator, *CA* community ambulator, *L* left, *R* right, *Ext* extension, *Abd* abduction, *+* indicates hyperextension, *Dors* dorsiflexion, *Plant* plantarflexion, *bi* bilaterally, *uni* unilaterally

^a^Unilaterally

^b^Compensation for limb length discrepancy

Nineteen children were community ambulators, 11 with no need for a wheelchair and eight with the need for a wheelchair for longer distances outdoors, and five children were household ambulators (Table 2) [20].

The strength of the lower limb muscles was tested manually according to a 0–5 graded scale [21], with 5 indicating normal strength to grade 0 indicating the absence of muscle strength. Hip extension and hip abduction ranged between grades 4 and 5, knee extension between grades 0 and 5, and ankle dorsiflexion and plantarflexion between grades 0 and 5 (Table 2).

Hip and knee flexion contractures were defined as ≥10º from a neutral joint position, and plantarflexion contractures as less than a neutral position. Knee hyperextension was defined as >10º from a neutral position. Flexion contractures uni- or bilaterally were seen in the ankle in 12 children, in the knee in nine and in the hip in six children. Knee hyperextension uni- or bilaterally was seen in six children (Table 2). Assessment of muscle strength and joint range of motion were performed by the same physiotherapist (LV).

Twenty-two participants had undergone orthopaedic surgery, of which 19 had bony surgery and three soft tissue surgery (Table 2).

### O_2_-walking test and 6-minute walk test (6MWT)

Energy expenditure was measured as the rate of oxygen uptake during walking at a self-selected speed using the portable telemetric system Cosmed K4b^2^ (Cosmed Srl, Rome, Italy), which is a valid and reliable method to measure energy during walking in children with disabilities [15].

Functional exercise capacity was measured with the 6MWT, which is a valid and reliable method for assessing endurance and exercise tolerance in healthy children [22] and in children with disabilities [23]. The heart rate was measured with a polar heart rate monitor (Polar Electro Oy, Kempele, Finland).

Both the O_2_-walking test and the 6MWT were performed on the same 21-m oval walking track in the gait laboratory. The 6MWT was performed after completion of the O_2_ measurement.

Prior to the O_2_-walking test, the child was given verbal instructions and the opportunity to become familiar with the equipment. The test was standardised to 5 min rest, followed by 5 min walking at a self-selected speed and completed by 5 min rest after the test. During the two resting periods, the child was asked to sit quietly in a comfortable chair in order to achieve a stable heart rate.

Prior to the 6MWT, it was assured that the heart rate was at a stable resting steady-state level. Each child was instructed to cover as long a distance as possible in 6 min, without running. Encouragement such as “keep going” and “you are doing well”, as well as announcement of the time remaining, was given to the child with respect to the guidelines of the American Thoracic Society (ATS) [24].

### Data analysis

Oxygen consumption was measured in terms of the rate of oxygen consumed while walking, both as normalised by body weight per unit time (ml/kg/min) and as oxygen cost per metre walked during the test (ml/kg/m) [17]. Gross oxygen consumption and gross oxygen cost, including both resting and walking oxygen utilisation, were calculated from the last 2 min of the walking period and were normalised by body weight and the weight of orthoses and shoes. To calculate the net oxygen consumption, the oxygen consumption at rest was subtracted from the oxygen consumption during walking. The last 2 min of the first resting period and the last 2 min of the walking period were used to calculate the net non-dimensional consumption (NNconsumption) and cost (NNcost). The net non-dimensional values were calculated including parameters of body weight, leg length and the acceleration due to gravity [18]. The weight of orthoses and shoes were included in the equation. The 6MWT was analysed based on the distance walked and the mean walking velocity during the complete test. For both tests, the walking velocity was normalised to leg length and is presented as the normalised walking velocity (N walking velocity) [25].

### Statistical analysis

AMC1 was excluded from the statistical comparison with the other groups because of the low participant number, with only descriptive statistics reported as mean (SD). A one-way analysis of variance (ANOVA) was used to compare values between AMC2, AMC3 and TD children. To identify significant differences among means between groups, a post hoc analysis was performed using Fisher’s least significant difference (LSD) test. All statistical analyses were carried out using commercially available software (SPSS version 19.0). A *p*-value of ≤0.05 was considered to be statistically significant.

## Results

### O_2_-walking test

One child in AMC2 and one in AMC3 did not complete the test.

The gross oxygen consumption (ml/kg/min) ranged in AMC1 between 10.15 and 14.56, in AMC2 between 16.38 and 29.20, in AMC3 between 12.92 and 25.69, and in TD children between 11.51 and 24.94. The gross oxygen cost (ml/kg/m) ranged in AMC1 between 0.28 and 0.40, in AMC2 between 0.21 and 0.71, in AMC3 between 0.19 and 0.41, and in TD children between 0.16 and 0.43. The walking velocity (m/s) was in AMC1 0.61, ranged in AMC2 between 0.60 and 1.31, in AMC3 between 0.55 and 1.29, and in TD children between 0.75 and 1.45.

NNconsumption was higher in AMC2 and AMC3 than in the TD children, but not significantly. NNcost was higher in AMC2 and AMC3 than in the TD children. As analysed with Fisher’s LSD post hoc test, AMC2 had significantly higher NNcost than TD children. N walking velocity was lower in AMC2 than in AMC3 and the TD children, but not significantly (Table 3).

**Table 3:** Normalised oxygen consumption (NNconsumption), normalised oxygen cost (NNcost) and normalised walking velocity in AMC2, AMC3 and TD children during the O_2_-walking test

	AMC2 (*n* = 10) (mean, SD)	AMC3 (*n* = 9) (mean, SD)	TD (*n* = 25) (mean, SD)	*p*-Value	AMC2–AMC3 *p*-Value	AMC2–TD *p*-Value	AMC3–TD *p*-Value
NNconsumption	0.156 (0.053)	0.159 (0.070)	0.123 (0.028)	0.055	–	–	–
NNcost	0.455 (0.193)	0.398 (0.146)	0.308 (0.069)	**0.007**	0.319	**0.002**	0.067
N walking velocity	0.361 (0.072)	0.401 (0.093)	0.403 (0.050)	0.223	–	–	–

*N* normalised, *AMC* arthrogryposis multiplex congenita, *TD* typically developing children

In AMC1, the NNconsumption mean (SD) was 0.082 (0.008), the NNcost mean (SD) was 0.385 (0.031) and the N walking velocity mean (SD) was 0.214 (0.005).

### 6MWT

The walking velocity (m/s) ranged in AMC1 between 0.82 and 0.93, in AMC2 between 0.66 and 1.53, in AMC3 between 0.81 and 1.88, and in TD children between 0.96 and 1.98.

The walked distance differed significantly between AMC2, AMC3 and the TD children. As analysed with Fisher’s LSD post hoc test, TD children walked a significantly longer distance than AMC2 and AMC3. N walking velocity differed significantly between AMC2, AMC3 and the TD children. As analysed with Fisher’s LSD post hoc test, AMC2 had significantly lower N walking velocity than TD children (Table 4).

**Table 4:** Walked distance and normalised walking velocity in AMC2, AMC3 and TD children during the 6-minute walk test (6MWT)

	AMC2 (*n* = 11) (mean, SD)	AMC3 (*n* = 10) (mean, SD)	TD (*n* = 25) (mean, SD)	*p*-Value	AMC2–AMC3 *p*-Value	AMC2–TD *p*-Value	AMC3–TD *p*-Value
Distance (m)	402.7 (108.0)	476.8 (126.1)	565.1 (110.0)	**0.001**	0.141	<**0.001**	**0.043**
N walking velocity	0.435 (0.030)	0.501 (0.100)	0.564 (0.073)	**0.001**	0.088	**<0.001**	0.055

*m* metres, *N* normalised, *AMC* arthrogryposis multiplex congenita, *TD* typically developing children

AMC1 walked a mean (SD) distance of 312.0 (19.7) m and had a N walking velocity mean (SD) of 0.304 (0.015).

## Discussion

The results in this study confirm that the energy effort during walking at a self-selected speed is increased in children with AMC. This was seen as higher NNconsumption in AMC2 and AMC3 than in the TD children, as well as a higher NNcost in AMC2 and AMC3 compared to TD children, which indicates a less efficient gait in children with AMC. Higher N walking velocity was found in all groups during the 6MWT compared to the O_2_-walking test, which was expected, as the instructions on walking speed differed between the two tests. Another explanation is that wearing the mask during the O_2_-walking test may have reduced the visual field, possibly influencing walking velocity. In this study, we included children aged 5–17 years, and, therefore, we chose to normalise the data with the net non-dimensional scheme, which has been recommended when including children of different ages [18].

Since orthoses aim to stabilise joints, they influence both gait pattern and walking function. We, therefore, divided the participants into groups according to their orthoses, as has been described in a previous study [11]. Among the AMC groups, the lowest NNcost was found in AMC1. We could not, however, confirm this observation statistically due to the low participant number. Children in AMC1 required above-knee orthotic solutions with locked knee joints and were totally dependent on orthoses to achieve walking ability. This group had the lowest NNconsumption of all groups, including TD children, which is probably related to their low walking velocity to decrease physical exertion. In normal gait, the knee flexes during the swing phase [26], which is not possible in a locked KAFO. Compensatory movements such as pelvic hiking or circumduction of the leg [26] are, therefore, necessary. In persons with knees immobilised with a cast, reduced walking speed and increased oxygen cost have been reported [27]. In AMC1, it was possible to maintain the oxygen consumption at an acceptable level by reducing walking velocity, which contributes to an increase in oxygen cost. The highest NNcost was found in AMC2, of whom all needed orthoses and all except two children were community ambulators.

Joint contractures, which are the main characteristics in AMC, were frequently found in the ankle joint in AMC1 and in AMC2, but only in two children in AMC3. The children in AMC3, who use only shoes, reached walking speeds most similar to the TD children during the O_2_-walking test. This may be due to not only fewer joint contractures than the other AMC groups, but also to the good plantarflexor strength in all children in AMC3. All participants had good muscle strength in hip extensors and hip abductors, and only one child had a hip flexion contracture of 20°. The presence of hip flexion contracture <20° and active hip motion were reported to be important factors to maintain community or independent walking [5].

Walking distance is the main outcome measure in the 6MWT [24]. The children with AMC walked shorter distances than the TD children, wherein children in AMC1 walked the shortest distance, which reflects the difficulties in keeping up with peers and, therefore, making wheelchair use necessary and useful. The large variety in walked distance within the AMC2, AMC3 and TD groups, however, may partly be explained by age variations, as this parameter was not normalised. The adolescents in AMC1 ambulated indoors using orthoses with locked knee joints and used powered wheelchairs for transportation. This is in accordance with Murray and Fixsen [28], who reported that adolescents felt more integrated into the community in a wheelchair than walking with crutches and orthoses. To meet each individual’s requirements, we recommend a combination of wheelchair and orthoses use for children who use orthoses for walking function. The requirement of walkers has been described in children walking with KAFOs, and often with forearm support instead of standard walker grips [9]. Furthermore, limited ambulation due to poor protective responses of the upper extremity has been reported [9]. Fifteen of 24 children in our study had upper limb involvement, of which two had locked KAFOs bilaterally, though none used a walking aid. This is believed to be due to the stable orthosis construction offering the children sufficient stability to walk without an external walking aid. Another important treatment goal is to improve biomechanical alignment with adequate orthoses and footwear combinations [29]. In our participants, heel height was adjusted in the orthoses and/or the shoes, when required, for each child’s contracture.

While lower ambulatory and physical activity has been reported in youths with AMC compared to TD youths [19], this is, to our knowledge, the first study on physical effort in children with AMC. The small number of participants and the heterogeneity in this study are limitations. Simultaneously, they reflect the rarity and complexity of this condition. A walking test of longer duration or walking at higher or fixed speeds may have discriminated even more between the groups. This, however, may have further reduced the study population due to limited endurance, which has been reported in youths with AMC [19]. Nevertheless, the self-selected speed which was chosen in this study has been suggested to be the best simulation of how children with disabilities function in the community [15].

## Conclusion

Energy expenditure and functional exercise capacity were evaluated in children with AMC during walking in their habitual orthoses, ranging from KAFO with locked knee joints, to AFO, to shoes only. The energy effort during walking at a self-selected speed was higher in children with AMC than in TD children The children walking with open KAFOs or AFOs, most of whom were community ambulators, walked with almost the same N walking velocity as AMC children not using orthoses and TD children. Their NNcost, however, was higher, which indicates a less efficient gait in this group. The children walking with KAFO with locked knee joints were totally dependent on orthoses to achieve walking ability. They had the lowest N walking velocity of all groups, and reducing walking velocity is interpreted as a strategy to minimise exertion. During the exercise capacity test in the 6MWT, the children with AMC walked a shorter distance than the TD children, which reflects their difficulties in keeping up with peers.
